# Brain structures in the sciences and humanities

**DOI:** 10.1007/s00429-014-0857-y

**Published:** 2014-07-31

**Authors:** Hikaru Takeuchi, Yasuyuki Taki, Atsushi Sekiguchi, Rui Nouchi, Yuka Kotozaki, Seishu Nakagawa, Carlos Makoto Miyauchi, Kunio Iizuka, Ryoichi Yokoyama, Takamitsu Shinada, Yuki Yamamoto, Sugiko Hanawa, Tsuyoshi Araki, Hiroshi Hashizume, Yuko Sassa, Ryuta Kawashima

**Affiliations:** 1 Division of Developmental Cognitive Neuroscience, Institute of Development, Aging and Cancer, Tohoku University, 4-1 Seiryo-cho, Aoba-ku, Sendai, 980-8575 Japan; 2 Division of Medical Neuroimaging Analysis, Department of Community Medical Supports, Tohoku Medical Megabank Organization, Tohoku University, Sendai, Japan; 3 Department of Radiology and Nuclear Medicine, Institute of Development, Aging and Cancer, Tohoku University, Sendai, Japan; 4 Department of Functional Brain Imaging, Institute of Development, Aging and Cancer, Tohoku University, Sendai, Japan; 5 Human and Social Response Research Division, International Research Institute of Disaster Science, Tohoku University, Sendai, Japan; 6 Smart Ageing International Research Center, Institute of Development, Aging and Cancer, Tohoku University, Sendai, Japan; 7 Japan Society for the Promotion of Science, Tokyo, Japan

**Keywords:** Academia, Sciences, Humanities, Area of study, Voxel-based morphometry, Cognitive functions

## Abstract

The areas of academic interest (sciences or humanities) and area of study have been known to be associated with a number of factors associated with autistic traits. However, despite the vast amount of literature on the psychological and physiological characteristics associated with faculty membership, brain structural characteristics associated with faculty membership have never been investigated directly. In this study, we used voxel-based morphometry to investigate differences in regional gray matter volume (rGMV)/regional white matter volume (rWMV) between science and humanities students to test our hypotheses that brain structures previously robustly shown to be altered in autistic subjects are related to differences in faculty membership. We examined 312 science students (225 males and 87 females) and 179 humanities students (105 males and 74 females). Whole-brain analyses of covariance revealed that after controlling for age, sex, and total intracranial volume, the science students had significantly larger rGMV in an anatomical cluster around the medial prefrontal cortex and the frontopolar area, whereas the humanities students had significantly larger rWMV in an anatomical cluster mainly concentrated around the right hippocampus. These anatomical structures have been linked to autism in previous studies and may mediate cognitive functions that characterize differences in faculty membership. The present results may support the ideas that autistic traits and characteristics of the science students compared with the humanities students share certain characteristics from neuroimaging perspectives. This study improves our understanding of differences in faculty membership which is the link among cognition, biological factors, disorders, and education (academia).

**Electronic supplementary material:**

The online version of this article (doi:10.1007/s00429-014-0857-y) contains supplementary material, which is available to authorized users.

## Introduction

The area of academic interest (sciences or humanities) and area of study are known to be associated with a number of factors. For example, men tend to outnumber women in careers that involve typical science-based thinking (such as engineering) (Billington et al. 2007; Lawrence 2006) and are less commonly involved in careers involving social skills (such as those typical of humanities majors). There may be a number of complex explanations for this phenomenon and it is a matter of controversy (Billington et al. 2007). Further, autistic traits have been shown to be associated with superior skill in areas that involve typical science-based thinking, and family members of those with autism are more prevalent in these fields (Baron-Cohen et al. 1998; Wheelwright and Baron-Cohen 2001).

Members or students of science faculties or faculties that involve typical science-based thinking are known to have a number of characteristics that have also been associated with autistic traits, as described below. These members or students have rare immune disorders (Temple 1990), more language-related problems in childhood (Temple 1990), higher visuospatial abilities (Billington et al. 2007), lower ability to read another’s feelings (Billington et al. 2007), lower empathizing (drive to identify others’ mental states to predict their behavior and respond with an appropriate emotion), and higher systemizing (drive to analyze a system in terms of the rules that govern the system to predict its behavior) (Billington et al. 2007; Wakabayashi et al. 2006; Wheelwright et al. 2006a) than the students of humanities or languages.

As described above, the area of study is associated with a wide range of cognitions, and research on these differences can improve our understanding of the links between cognition, biological factors, disorders, and education (academia). However, despite the unique importance of the area of study, the neural correlates of differences between faculty members are unknown.

Regional gray matter volume (rGMV) is known to be increased in areas in the medial prefrontal cortex (mPFC), anterior cingulate cortex (ACC), and certain parts of the dorsolateral prefrontal cortex (DLPFC) in autistic subjects (for meta-analyses, see Via et al. 2011; Yu et al. 2011), though these meta-analytic findings may have to be taken cautiously, given the larger motion in autistic subjects (Nordahl et al. 2008). Our previous study with a large sample size showed that less rGMV in all or some of these areas seems to be associated with enhanced empathizing as well as reduced systemizing (Takeuchi et al. 2014). Partly consistently, a study from another laboratory (Lai et al. 2012) showed that decreased rGMV in ACC and mPFC was associated with a smaller discrepancy between systemizing and empathizing (systemizing minus empathizing). In this previous study, however, this association seemed to be driven mainly by the association between less systemizing and decreased rGMV in ACC and mPFC. On the other hand, a meta-analysis has shown rGMV reduction in both the amygdala and hippocampus of individuals with autism (Via et al. 2011), whereas another meta-analysis has shown rGMV reduction in the hippocampus of individuals with autism, as well as rGMV reduction in both the amygdala and hippocampus in individuals with Asperger syndrome (Yu et al. 2011). In addition, the hippocampus is known to be associated with certain spatial cognitions (Maguire et al. 2000). We therefore hypothesized that increased rGMV in the areas around the anterior brain regions including the mPFC and reduced volume in the hippocampus and/or the amygdala are associated with science students as was the case of autistic subjects.

The purpose of this study was to test these hypotheses and to reveal differences in rGMV and rWMV between the science and humanities students. We therefore compared rGMV and rWMV of 312 university science students and 179 university humanities students using voxel-based morphometry (VBM) (Good et al. 2001).

Functional imaging studies and structural studies have both advantages and disadvantages, but the findings from the two methods complement each other. Structural imaging studies are especially useful for investigating the anatomical correlates of personal characteristics involving a wide range of cognitions such as an individual’s area of study, which seems to be associated with a wide range of cognitions. Unlike functional magnetic resonance imaging (fMRI) studies, the results of structural imaging studies are not limited to the specific regions engaged in the specific tasks or stimuli during scanning.

## Methods

### Subjects

Four-hundred and ninety healthy, right-handed individuals participated in this study as part of our ongoing project to investigate the associations among brain imaging, cognitive functions, aging, genetics, and daily habits (Sassa et al. 2012; Takeuchi et al. 2011c, d, 2012a, 2013b; Taki et al. 2011a, b). All were college students from Tohoku University in Japan. The sciences group consisted of 312 participants, of whom 225 were male and 87 female. The humanities group consisted of 179 participants, of whom 105 were male and 74 were female. All the participants were between 18 and 25 years of age. Some of the subjects who took part in this study also became subjects of our intervention studies (psychological data and imaging data recorded before the intervention were used in this study) (Takeuchi et al. 2011b, e, 2014). Psychological tests and MRI scans not described in this study were performed together with those described in this study. The mean age of subjects was 20.2 years [standard deviation (SD) 1.5]. All subjects had normal vision and none had a history of neurological or psychiatric illness. Handedness was evaluated using the Edinburgh Handedness Inventory (Oldfield 1971).

### Determination of the area of study

Participants reported their undergraduate area of study and were then classified as science or humanities students. The sciences included the Faculty of Science, Faculty of Engineering, Faculty of Pharmaceutical Sciences, and Faculty of Agriculture. The humanities included the Faculty of Arts and Letters, Faculty of Education, Faculty of Law, and Faculty of Economics. Each faculty has one or multiple measures (for example, Faculty of Science has Department of Mathematics, Department of Physics and so on) and each student belongs to one measure.

How faculties are classified when differences in the area of study are studied varies substantially on the basis of the studies. Some studies have focused on faculties/majors related to mathematics and verbal areas (Temple 1990), while others have included both the biological sciences and physical sciences as part of the sciences but have divided the non-science majors into two categories (Baron-Cohen et al. 2001), and yet other studies have indicated differences between (a) physics and engineering and (b) math and chemistry among science majors (Focquaert et al. 2007). We basically followed the classification of Wakabayashi et al. (2006) because of the similar cultural background. However, we excluded medical and dental students from the present study because of their apparent academic performance difference as well as because of their different characteristics compared with the rest of the science and humanities students (after graduation, almost all medical and dental students become experts such as doctors, nurses, and dentists). With this division, the non-medical and non-dental faculties of our university could easily be divided into two, and these classifications also matched the subjects required for the Tohoku University entrance examination. Students with sciences and humanities majors can be considered to have almost equivalent academic performance on average.

In Japan, before completing the entrance examinations for each university and faculty, students normally take part in a common examination known as the “National Center Test for University Admissions.” Rough standard scores for the National Center Test for University Admissions that are required to pass the entrance examinations at Tohoku University differ slightly among faculties, but the average score of those in the science and humanities faculties are estimated to be almost the same (http://www.toshin.com/centerlist/mainfrm_np.php?pref=%E5%AE%AE%E5%9F%8E%E7%9C%8C#0). This suggests that the scholastic performances of science and humanities students are almost the same.

### Psychological outcome measures

Neuropsychological tests and questionnaires were administered. We analyzed cognitive functions specifically believed to be associated with the area of study as well as control basic cognitive functions that are believed to be irrelevant to the area of study.

Measures of control of basic cognitive functions believed to be irrelevant to the area of study are as follows: (a) A (computerized) digit span task, which is a working memory task (for details, see Takeuchi et al. 2011a). (b) The perception factor of the Tanaka B-type intelligence test (TBIT) (Tanaka et al. 2003) type 3B. This is a mass intelligence test used for third-year junior high school and older examinees. The perception speed factor of TBIT measures simple processing speed. In all subtests, the subjects have to solve as many problems as possible before a certain time (a few minutes). This factor involves three subtests: a displacement task [substitute a figure (9 figures) with a number (1–9) according to a model chart], identification vs. same–different judgments (Japanese kana characters; judge whether a pair of meaningless Japanese strings are the same), and marking figures [select forms identical to 3 samples from a series (sequence) of 8 different forms]. Measures of cognitive functions specifically believed to be associated with the area of study are as follows. (c) RAPM (Raven 1998), a non-verbal reasoning task. Non-verbal reasoning is theoretically linked to systemizing. (d) Spatial relation factor of TBIT measures spatial abilities to relate different things. In all subtests, the subjects have to solve as many problems as possible before a certain time (a few minutes). This factor involves three subtests: the maze test (trace a maze with a pencil from start to finish), counting cubes (count cubes piled up in a three-dimensional manner), and filling in figures (complete incomplete figures so that they are the same as the sample figures when rotated). (e) Reading comprehension task. This task was developed by Kondo et al. (2003). It involves eight sections of articles; each article has four questions and each question has five choices of answers. These questions are designed so that participants can determine the correct answers if they read the articles correctly. The subjects were asked to correctly answer as many questions as possible over 13 min. Similar to the reading comprehension task developed in Western countries, the score for this task has a significant positive correlation with the score for the reading span task. For more details on this test, such as how it was developed and its validity, please refer to Kondo et al. (2003). (f) SQ and EQ questionnaires. Japanese versions (Wakabayashi et al. 2007) of the SQ and EQ questionnaires (Baron-Cohen and Wheelwright 2004; Baron-Cohen et al. 2003) were administered. The EQ score was used as an index of empathizing, and the SQ score was used as an index of systemizing. Data for each cognitive measure were obtained from all or some of the subjects. The number of subjects from whom we obtained data for each cognitive measure is shown in Table 1.

**Table 1:** Psychological variables of the study participants

Measure	Sciences		Humanities		*P* value*
	Mean	SD	Mean	SD	
Age (*S* = 312, *H* = 179)	20.08	1.39	20.33	1.55	0.051
Digit span (*S* = 298, *H* = 161)	36.17	6.85	35.56	6.66	0.592
Perception speed factor of TBIT (*S* = 295, *H* = 161)	49.59	7.02	49.16	7.11	0.439
RAPM (*S* = 312, *F* = 179)	29.45	3.36	27.17	3.71	4.33 × 10^−11^
Spatial relation factor of TBIT (*S* = 298, *H* = 161)	43.57	4.73	41.40	4.91	9.34 × 10^−5^
Reading comprehension (*S* = 228, *H* = 114)	13.86	4.57	15.59	4.86	0.001
EQ (*S* = 311, *H* = 179)	28.38	9.29	32.01	9.84	0.001
SQ (*S* = 311, *H* = 179)	28.14	8.91	22.88	8.32	1.20 × 10^−8^

*S* science students, *H* humanities students

* *P* value of ANCOVA with additional covariates of age and sex (in case of the analysis of age, only sex was an additional covariate)

### Behavioral data analysis

The behavioral data were analyzed using the statistic software SPSS 22.0 (IBM SPSS Inc., Armonk, NY, USA). Differences in scores for the cognitive measures between the science and humanities students were analyzed using analysis of covariance (ANCOVA). Additional covariates for each analysis were age and sex. Results with a threshold of *P* < 0.05 were considered statistically significant in these analyses.

### Image acquisition

All MRI data acquisition was performed using a 3-T Philips Intera Achieva scanner. High-resolution T1-weighted structural images (T1WIs; 240 × 240 matrix, TR = 6.5 ms, TE = 3 ms, FOV = 24 cm, slices = 162, in-plane resolution = 1 × 1 mm, slice thickness = 1.0 mm) were collected using a magnetization-prepared rapid gradient echo sequence.

### Preprocessing and analysis of structural data

Preprocessing of the structural data was performed using Statistical Parametric Mapping software (SPM8; Wellcome Department of Cognitive Neurology, London, UK) implemented in Matlab (Mathworks Inc., Natick, MA, USA). Using the new segmentation algorithm implemented in SPM8, T1-weighted structural images of each individual were segmented into six tissues. In this process, the gray matter tissue probability map (TPM) was manipulated from maps implemented in the software so that the signal intensities of voxels with (gray matter tissue probability of the default tissue gray matter TPM + white matter tissue probability of the default TPM) <0.25 became 0. When this manipulated gray matter TPM is used, the dura mater is less likely to be classified as gray matter (compared with when the default gray matter TPM is used), without other substantial segmentation problems. In this new segmentation process, default parameters were used, except that affine regularization was performed with the International Consortium for Brain Mapping template for East Asian brains. We then proceeded to the diffeomorphic anatomical registration through exponentiated lie algebra (DARTEL) registration process implemented in SPM8. In this process, we used DARTEL import images of the six gray matter TPMs from the above-mentioned new segmentation process. First, the template for the DARTEL procedures was created using imaging data from 63 subjects who participated in an experiment in our laboratory (Takeuchi et al. 2011a). Next, using this existing template, the DARTEL procedures were performed for all of the subjects in the present study. In these procedures, default parameter settings were used. The resulting images were spatially normalized to the Montreal Neurological Institute (MNI) space to give images with 1.5 × 1.5 × 1.5 mm^3^ voxels. In addition, we performed a volume change correction (modulation) by modulating each voxel with the Jacobian determinants derived from spatial normalization, which allowed us to determine regional differences in the absolute amount of brain tissue (Ashburner and Friston 2000). Subsequently, all images were smoothed by convolving them with an isotropic Gaussian kernel of 10 mm full width at half maximum (FWHM) for the reasons described below.

### Statistical analysis

We investigated group differences in rGMV and rWMV between the science and humanities students. Statistical analyses of imaging data were performed with VBM5 software, an extension of SPM5 for the reasons described below.

In the analyses of rGMV and rWMV, we included only voxels that showed rGMV and rWMV values of >0.05 in all subjects. The primary purpose for using this type of threshold is to cut the periphery of the GM and WM area and effectively limit the area for analysis to areas likely to be GM and WM. Voxels outside the brain areas are more likely to be affected by signals outside the brain through smoothing. The GM and WM value threshold of 0.05 is a widely used value that has been reported in numerous previous VBM studies (Beal et al. 2007; Focke et al. 2008; Mueller et al. 2006; Nauchi and Sakai 2009; Schaufelberger et al. 2007; Takeuchi et al. 2010a, b; White et al. 2003).

In whole-brain ANCOVAs, membership of the different faculties was used as a group factor (using the full factorial option of SPM) to determine differences in rGMV and rWMV between the science and humanities students. The analyses were performed considering sex, age, and total intracranial brain volume (total GM volume + total WM volume + total cerebrospinal fluid volume) as additional covariates. For each covariate, the “overall mean” option was used for centering. All of these covariates were modeled so that each covariate had a common relationship with rGMV and rWMV across the different groups. The group differences in rGMV and rWMV were assessed using t contrasts.

The statistical significance level was set at *P* < 0.05, corrected at the non-isotropic adjusted cluster level (Hayasaka et al. 2004) with an underlying voxel level of *P* < 0.0025. In this non-isotropic cluster size test of random field theory, a relatively higher cluster-determining threshold combined with high smoothing values of more than six voxels leads to appropriate conservativeness in real data. With high smoothing values, an uncorrected threshold of *P* < 0.01 seems to lead to anticonservativeness, whereas that of *P* < 0.001 seems to lead to slight conservativeness (Silver et al. 2010). We used the VBM5/SPM5 version of this test and a smoothing value of 10 mm. This is because a previous validation study of this test using a real dataset (Silver et al. 2010) showed that the conditions of this non-isotropic adjusted cluster size test are very limited and depend on the smoothness of the data, as described above. However, there are substantial differences in the way that SPM8 and SPM5 estimate actual FWHM in the areas analyzed, and this directly affects the cluster test threshold. Therefore, regardless of whether SPM5 or SPM8 is appropriate, our view is that the conditions for this non-isotropic adjusted cluster size test shown by the previous study (Silver et al. 2010) are no longer guaranteed in SPM8, because they are different analyses and produce substantially different results. We used a 10 mm FWHM instead of a 12 mm FWHM, which was recommended in the previous study (Silver et al. 2010), because the normalized volume image from the DARTEL procedure seems to be smoother and because 10 mm FWHM seems to be sufficient for achieving actual smoothness in the analyzed areas that can be acquired when the recommended smoothing value (12 mm FWHM) is used for segmented VBM images from previous versions.

## Results

### Behavioral data

Table 1 shows the average and SD of the Digit span (working memory), perception speed factor of TBIT (simple PS), RAPM (non-verbal reasoning), spatial relation factor of TBIT (spatial abilities), a reading comprehension task, and EQ (empathizing) and SQ (systemizing) scores of the science and humanities students. ANCOVAs revealed that after adjusting for the effect of age and sex, the science students had significantly higher RAPM, spatial relation factor of TBIT, and SQ scores than the humanities students, as well as significantly lower reading comprehension and EQ scores. No significant differences were observed in basic cognitive ability (simple PS; the score of perception speed factor of TBIT), suggesting good control for basic cognitive ability between the science and humanities students. Further, no significant differences were observed in working memory (digit span) between the science and humanities students. The differences in EQ and SQ observed between the science and humanities students are consistent with the results of previous studies (Billington et al. 2007; Focquaert et al. 2007; Wakabayashi et al. 2006). Processing speed and working memory are important factors underlying general academic attainment (Rohde and Thompson 2007); thus, lack of differences in scores of these tasks between the science and humanities students further suggest the lack of difference of general abilities for academic achievement between these groups in Tohoku University.

### Differences in rGMV between science and humanities students

**Fig. 1:**
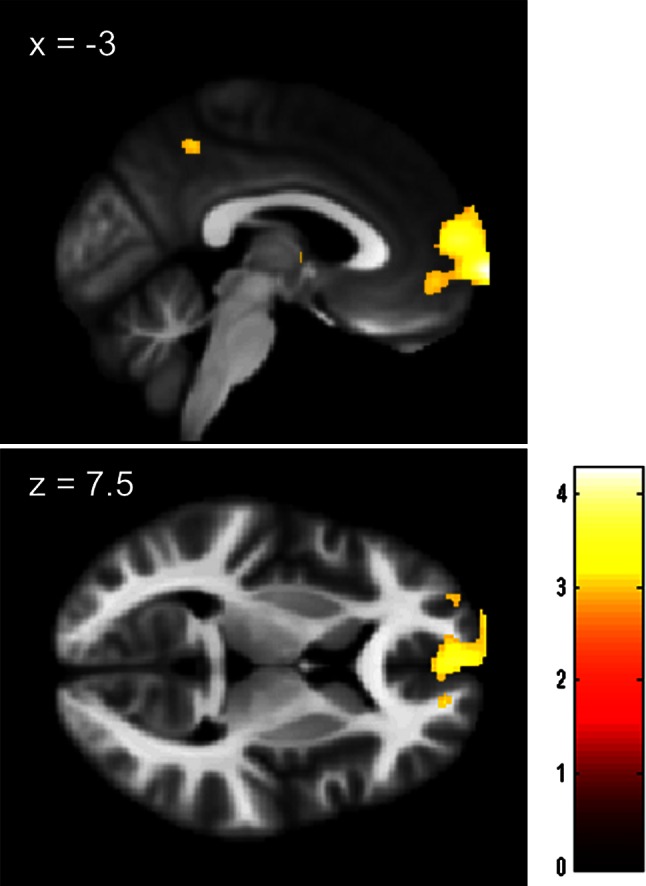
Maps showing larger rGMV in science students than in humanities students. The results are presented at a threshold of *P* < 0.0025, uncorrected, on a skull-stripped image created from the averaged normalized T1-weighted structural images of a portion of the subjects included in this study. The region of significant difference is visible in an anatomical cluster that spreads through mPFC and the frontopolar area. The intensity of the *color* represents the *T* score

**Fig. 2:**
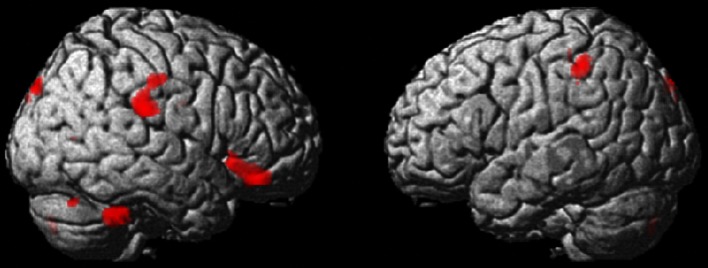
Maps showing the tendency toward larger rGMV in humanities students than in science students. The results are presented at a threshold of *P* < 0.0025, uncorrected. The region where a tendency toward a difference was visible in an anatomical cluster that spreads through the orbital part of the right inferior frontal gyrus. A similar tendency was observed in the right supramarginal gyrus

ANCOVA revealed that after adjusting for the effects of age, sex, and TIV, an anatomical cluster that included the frontopolar area and mPFC was found to have significantly larger rGMV in the science students [MNI coordinates of the peak, *x*, *y*, *z* = −2, 74, −3; *t* value of the peak = 4.26; corrected cluster *P* value (see “Methods” for details) = 0.035, Fig. 1]. The same analysis revealed marginally significantly larger rGMV in an area of the orbital part of the right inferior frontal gyrus in the humanities students (MNI coordinates of the peak, *x*, *y*, *z* = 54, 36, −18; *t* value of the peak = 3.77; corrected cluster *P* = 0.066, Fig. 2). A tendency toward larger rGMV in an anatomical cluster mainly concentrated around the right supramarginal gyrus (MNI coordinates of the peak, *x*, *y*, *z* = 72, −28, 25; *t* value of the peak = 3.53; raw cluster size of 1,478 mm^3^, under the voxel-level cluster-determining threshold of *P* < 0.0025, Fig. 2) was also observed in the humanities students. There were no other significant results. However, the results were weak and not robust.

### Differences in rWMV between science and humanities students

**Fig. 3:**
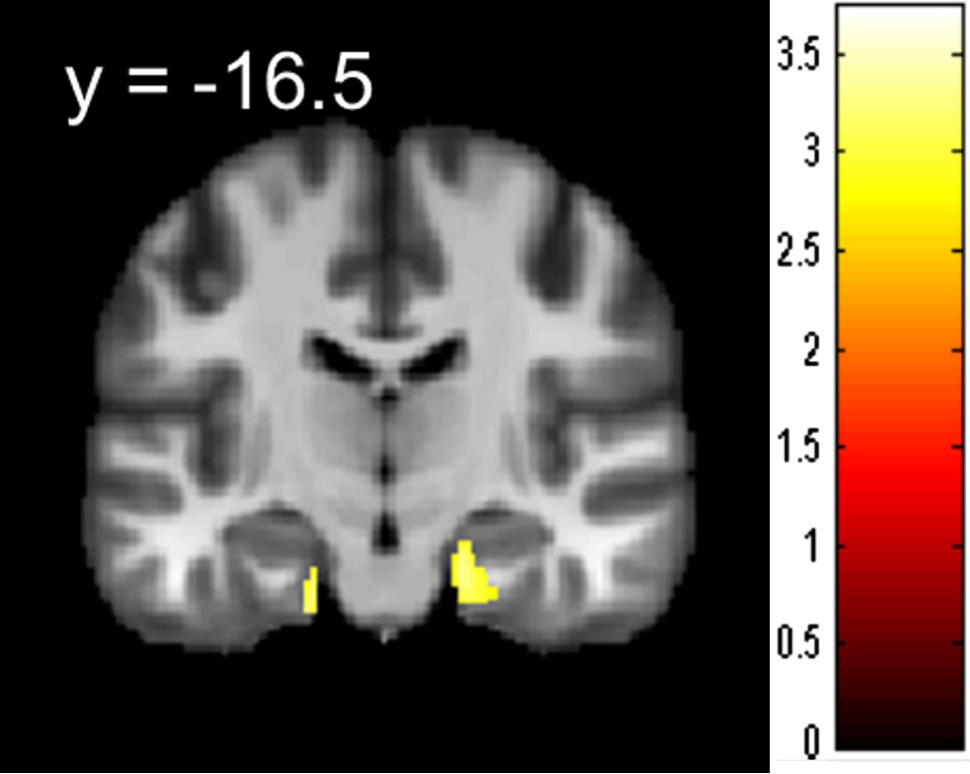
Maps showing larger rWMV in humanities students than in science students. The results are presented at a threshold of *P* < 0.0025, uncorrected, on a skull-stripped image created from the averaged normalized T1-weighted structural images of a portion of the subjects included in this study. The region of significant difference is visible in an anatomical cluster that is mainly concentrated around the right hippocampus. The intensity of the *color* represents the *T* score

ANCOVA revealed that after adjusting for the effects of age, sex, and TIV, the humanities students had significantly larger rWMV in an anatomical cluster mainly concentrated in the right hippocampus (MNI coordinates of the peak, *x*, *y*, *z* = 26, −28, −29; *t* value of the peak = 3.73; corrected cluster *P* = 0.018, Fig. 3). There were no other significant findings.

## Discussion

To the best of our knowledge, this is the first study to investigate differences in rGMV and rWMV between university science and humanities students. We newly revealed that compared with the humanities students, the science students have larger rGMV in an anatomical cluster that includes mPFC and the frontopolar area as well as lower rWMV in an anatomical cluster mainly concentrated in the right hippocampus.

Our hypothesis of structural differences in science students was confirmed, to some extent, and the present results with science students are similar to those of previous meta-analysis studies of individuals with autism (Via et al. 2011; Yu et al. 2011). However, rWMV, rather than rGMV, of the hippocampus was reduced in science students.

The increased rGMV observed in the science students may be associated with lower empathizing, but the higher visuospatial ability of these students may also be associated with this structural characteristic. mPFC is functionally known to be associated with assessing the psychological attributes of a person, regardless of whether it is oneself (for a review, see Amodio and Frith 2006; Christoff and Gabrieli 2000) or other (Amodio and Frith 2006). Our previous study of subjects with similar characteristics showed that the reduction in rGMV in the mPFC was associated with higher empathizing (Takeuchi et al. 2014). Nevertheless, the analyses suggested that it is possible that rGMV correlates with empathizing, and the difference between science and humanities students overlapped substantially in mPFC. However, this is not clear from the present stringent statistical threshold that was applied to the whole brain (see Supplemental Methods, Supplemental Results, and Supplemental Figs. 1 and 2 for details). Indeed, an association between better social or self-related cognitive functioning and lower rGMV in regions around areas close to mPFC has been clearly demonstrated in young adults (Banissy et al. 2012; Takeuchi et al. 2011c, 2013a) and may reflect advanced cortical developmental thinning (Sowell et al. 2003) and thus improved functioning (Kanai and Rees 2011). Autistic subjects, who are characterized by higher systemizing and lower empathizing, have larger rGMV in this area (Yu et al. 2011). However, non-verbal visuospatial reasoning ability is positively correlated with rGMV in this area among older adults (Gong et al. 2005). In our previous studies (Takeuchi et al. 2013c, 2014), we suggested that there is a trade-off between empathizing and systemizing in the brain, and structural volume in a wide range of areas show positive/negative correlation with empathizing and negative/positive correlation with systemizing at once. However, it should be noted that despite these correlations, empathizing and systemizing tend to show little correlation when sex is corrected (Takeuchi et al. 2014; Wakabayashi et al. 2007; Wheelwright et al. 2006b). On the other hand, a previous study revealed the positive correlation between visuospatial reasoning ability and rGMV of mPFC (Gong et al. 2005). rGMV of mPFC was also positively correlated with spatial ability in the present study (see Supplemental Methods and Supplemental Results for details). It is probable that the difference in rGMV in this area may represent a certain form of trade-off between cognitive functions related to social interactions and other types of cognitions.

The common neural and cognitive characteristics of science students and autistic subjects may be associated to a greater or lesser extent, but they may also partly be ascribed to fetal testosterone levels. These levels are positively correlated with autistic traits (Auyeung et al. 2009), systemizing (Auyeung et al. 2006), language difficulties (Whitehouse et al. 2010), and spatial abilities (Grimshaw et al. 1995) but are negatively correlated with empathizing and an ability to read others’ minds (Chapman et al. 2006), which are characteristics of science students, as described in “Introduction”. Finally, a recent study showed that a higher fetal testosterone level, along with those of other hormones, is associated with a later diagnosis of autism (Baron-Cohen et al. 2014),which is characterized by higher systemizing, lower empathizing, and language difficulties (Baron-Cohen 2003). However, certain associations are considered to show a non-linear relationship (too high and too low fetal testosterone levels have similar effects) and are thus complex (Puts et al. 2008). Fetal testosterone exposure is also associated with immune functioning (Martin 2000). Thus, fetal testosterone levels may have mediated the structural characteristics observed in the science students. A meta-analysis has shown rGMV reduction in both the amygdala and hippocampus of individuals with autism (Via et al. 2011), whereas another meta-analysis has shown rGMV reduction in the hippocampus of individuals with autism, as well as rGMV reduction in both the amygdala and hippocampus in individuals with Asperger syndrome (Yu et al. 2011). However, fetal testosterone level is “positively” correlated with rGMV in areas extending to both the hippocampus and amygdala (Lombardo et al. 2012).

The larger rWMV in the right hippocampus observed in the humanities students may be associated with inferior abilities with respect to a certain form of spatial cognition. As described in “Introduction”, a reduction in hippocampal rGMV in autistic subjects is one of the most robust findings of structural studies of autistic subjects (Via et al. 2011; Yu et al. 2011). Our present finding in relation to the right hippocampus is congruent with this reduction given that autistic traits are associated with the sciences, as described in “Introduction”. In the present study, we identified negative associations between ASC-related variables and hippocampal volume. However, the fetal testosterone level, which is positively correlated with autistic traits, is actually positively correlated with rGMV in the amygdaloid–hippocampal area among normal subjects (Lombardo et al. 2012). In this previous study, there were positive associations between ASC-related variables and hippocampal volume. A wide range of evidence has suggested that spatial cognitive abilities are considered to show an inverted U relationship with fetal testosterone levels. In other words, excessively high or low levels of exposure lead to reduced functioning (Puts et al. 2008). Considering that the hippocampus volume is associated with spatial cognitive abilities and testosterone increases the hippocampus structure and spatial cognitive abilities in certain conditions (Roof and Havens 1992), we speculate that there is a possibility that, while usually higher level of fetus testosterone levels lead to higher spatial abilities and larger hippocampal volume, too high levels of fetal testosterone may lead to the reduced hippocampal volume as is the case of the spatial abilities (Puts et al. 2008). Considering our study dealt with a non-clinical sample, it might be expected to show increased rGMV in the hippocampus area in science students. In other words, normal science students may be more likely to show moderately high testosterone levels that should lead to an increased hippocampal volume or function in the above-mentioned model. In fact, our rGMV analysis did show a tendency toward larger rGMV in the science students in that area (*T* value of the peak = 3.38), and the supplemental analysis of regional gray matter density (rGMD; the proportion of gray to all tissue types within a region) revealed significantly larger rGMD in the right hippocampus in the science students (*P* < 0.05, corrected at the cluster size level using the methods same as those for rGMV and rWMV analyses). Thus, the reduced rWMV observed in this area among the science students may be a result of the increase in rGMV in this small area and may not necessarily reflect facilitation of function in this area, but this is highly speculative. The hippocampus structure is associated with a number of seemingly distinct cognitions and is known to be reduced in a fairly wide range of clinical disorders (Geuze et al. 2004), increased after extensive learning in medical faculties (Draganski et al. 2006), and increased in taxi drivers, who may be viewed as experts with respect to spatial navigation and memory (Maguire et al. 2000). Thus, it is difficult to infer specific cognitions from the structures in this area. However, in this study, rWMV in the hippocampus was negatively correlated with spatial ability (see Supplemental Methods and Supplemental Results for details). Considering all of these findings, the increased rWMV in the right hippocampus of the humanities students may reflect lower spatial cognitive ability among these students.

The present results may support the ideas that autistic traits and characteristics of science students compared with humanities students share certain characteristics from neuroimaging perspectives. As described in “Introduction”, certain cognitive and physiological characteristics of autistic subjects are also observed in science students, but not humanities students. The larger rGMV in the mPFC area observed in the science students corresponds to the results of previous meta-analyses that have shown autistic subjects to have larger rGMV in this area. When combined with the meta-analysis finding of rGMV in autistic subjects, this may validate the idea that autistic subjects and science students share certain characteristics (Baron-Cohen et al. 2001). However, as was noted earlier, the hippocampal structural changes appear to require a more complex interpretation.

This study had a few limitations. One was common to almost all studies that investigated differences in the area of study (Focquaert et al. 2007; Temple 1990; Wakabayashi et al. 2006), i.e., we recruited subjects from a single university. Limited sampling of the full range of intellectual abilities is a common hazard in these studies. Whether our findings would also hold across the full range of population samples as well as a normal distribution must be determined with larger and more representative samples. Further, although we recruited as much as 491 subjects, it is becoming increasingly clear that structural studies of certain cognitions tend to require a large sample size (Takeuchi et al. 2012b, 2013a). With a much larger sample size, we might be able to find significant differences in some hypothesized areas, such as the inferior frontal gyrus, inferior parietal lobule to the supramarginal gyrus, and superior temporal sulcus, and provide a definitive answer with respect to the hypothesis regarding these areas.

In summary, differences in the area of study (sciences or humanities) are known to be associated with a wide range of cognitions, physiological factors, and autistic traits. We have revealed for the first time that the difference in the area of study is also visible as differences in rGMV of mPFC and the frontopolar area as well as in rWMV of the right hippocampus. Meta-analyses have shown that structures in these areas are altered in autistic subjects, and we suggest that cognitive differences between science and humanities students are at least partly associated with the volumetric properties of these areas. This study further improves our understanding of the differences in the area of study and the links between cognition, biological factors, disorders, and education (academia).

## Electronic supplementary material

Below is the link to the electronic supplementary material.

Supplementary material 1 (DOCX 660 kb)

Supplementary material 2 (TIFF 2953 kb)

Supplementary material 3 (TIFF 2953 kb)
