# Supplementary material for: Brain structures in the sciences and humanities
Source: Brain Struct Funct. 2014 Jul 31;220(6):3295–305. doi: 10.1007/s00429-014-0857-y (PMC4575694; doi:10.1007/s00429-014-0857-y)
Supplement: Supplementary file 1 — Supplementary material 1 (DOCX 660 kb) [file 429_2014_857_MOESM1_ESM.docx]

**Supplementary online material**

**Supplemental Methods**

**Post-hoc investigation of the association between the regional gray matter volume (rGMV) of the medial prefrontal cortex (mPFC) and empathizing.** We examined the negative association between rGMV of mPFC and empathizing in young adults, which has been suggested in previous studies, including our own ([Banissy et al. 2012](#_ENREF_1); [Takeuchi et al. 2014](#_ENREF_5)). Although our present and previous studies share a substantial number of subjects, many factors differed between these studies, such as the sample size, sample selection, and preprocessing methods (VBM2 vs. SPM8). Therefore, we first performed a post-hoc multiple regression analysis. For this analysis, we calculated the mean rGMV value for the significant cluster in mPFC which was identified in the analysis of differences of rGMV between science and humanities students. The dependent variable was the mean rGMV; the independent variables were sex, age, Raven’s Advanced Progressive Matrices test score, total intracranial volume (TIV), empathizing and systemizing (the same variables were examined in the previous study) ([Takeuchi et al. 2014](#_ENREF_5)). However, this analysis did not identify significant results. Therefore, we attempted to replicate our previous findings ([Takeuchi et al. 2014](#_ENREF_5)) in which empathizing negatively correlated with rGMV of mPFC using the present preprocessing methods and the entire project sample, which included the present subjects (students of science and humanities at the Tohoku University), students of other disciplines at the Tohoku University, and students at other universities as was the case in our previous study ([Takeuchi et al. 2014](#_ENREF_5)). This sample included 894 subjects (508 men, 386 women) with a mean age of 20.8 years (standard deviation [SD], 1.8). We also conducted this analysis with only the subjects in the present study and performed whole-brain multiple regression analyses. In these analyses, sex, age, Raven’s Advanced Progressive Matrices test score, TIV, empathizing and systemizing were the independent variables ([Takeuchi et al. 2014](#_ENREF_5)). The statistical threshold was same as that of the main text. We then compared the spatial distributions of rGMV correlates for the differences in empathizing between science and humanities students.

**Post-hoc investigation of the association between rGMV of mPFC and spatial abilities.** To confirm the association between rGMV of mPFC and spatial abilities, which was suggested in a previous study ([Gong et al. 2005](#_ENREF_2)), we performed a post-hoc multiple regression analysis. We calculated the mean rGMV value for the significant cluster in mPFC, which was identified in the analysis of differences of rGMVbetween science and humanities students. The dependent variable was the mean rGMV; the independent variables were sex, age, and the perception and spatial relation factor scores of the Tanaka B-type intelligence test (TBIT). Considering, the spatial factors related to intelligence have also been shown to be correlated with global brain gray matter volume ([Haier et al. 2009](#_ENREF_3)), we conducted the analyses with and without the additional covariate of TIV.

**Post-hoc investigation of the association between the regional white matter volume (rWMV) in the hippocampus and spatial abilities.** To confirm the association between hippocampal rWMV and spatial abilities, which was suggested in a previous study ([Maguire et al. 2000](#_ENREF_4)), we performed a post-hoc multiple regression analysis. We calculated the mean rWMV value for the significant cluster in the right hippocampus which was identified in the analysis of the differences or rWMV between science and humanities students. In this analysis, the dependent variable was the mean rWMV; the independent variables were sex, age, TIV, and the perception and spatial relation factor scores of TBIT.

**Supplemental Results**

**Post-hoc investigation of the association between rGMV of mPFC and empathizing.** This analysis failed to find a significant correlation between the mean rGMV of the significant cluster identified in the analysis of the difference between science and humanities students in mPFC and EQ score (empathizing, *P* > 0.1). Next, we investigated whether rGMV correlates of empathizing were distributed around mPFC in the whole-brain analysis. Using the entire study sample of this project, we identified a significant negative correlation between rGMV and empathizing in the medial to dorsomedial prefrontal cortex (MNI peak coordinates: x, y, z = 17, 41, 37; peak *t* value = 3.94; corrected cluster *P* value = 0.025, **Supplemental Fig. 1**). However, this significant cluster did not overlap with the significant cluster identified for the difference in rGMV of mPFC between science and humanities students, but did overlap slightly with the tendency of rGMV differences between science and humanities students (*P* < 0.0025, uncorrected, **Supplemental Fig.** **1**). Finally, the distribution of a weak tendency of a negative correlation between rGMV and empathizing (uncorrected, *P* < 0.05) and the distribution of a weak tendency of rGMV between science and humanities students (uncorrected *P* < 0.05) substantially overlapped (**Supplemental Fig. 2**). When only subjects in the present study (science and humanities students at the Tohoku University) were examined for rGMV correlates of empathizing, the relationship between empathizing and rGMV of mPFC was similar, but the relationship generally weakened and became insignificant, perhaps reflecting the reduced sample size. This suggests that it is possible rGMV correlates of empathizing and the differences between science and humanities students overlap substantially in mPFC. However, this relationship was not clear using the present stringent statistical threshold applied to the whole brain.

**Post-hoc investigation of the association between rGMV of mPFC and spatial abilities.** We identified a significant negative correlation between the mean rGMV of the significant cluster in mPFC which was identified in the analysis of the differences between science and humanities students and the spatial relation factor score of TBIT (*P* = 0.009, *t* = 2.611), although the associations were not significant when the TIV was included as a covariate (this might be because the volume of a wide range of other areas may show the same pattern).

**Post-hoc investigation of the association between the hippocampus rWMV and spatial abilities.** We identified a significant negative correlation between rWMV in this cluster in the right hippocampus which was identified in the analysis of the differences between science and humanities students and the spatial relation factor score of TBIT (*P* = 0.033, *t* = −2.142).

**Reference**

Banissy MJ, Kanai R, Walsh V, Rees G (2012) Inter-individual differences in empathy are reflected in human brain structure. Neuroimage 62:2034-2039.

Gong QY, Sluming V, Mayes A, Keller S, Barrick T, Cezayirli E, Roberts N (2005) Voxel-based morphometry and stereology provide convergent evidence of the importance of medial prefrontal cortex for fluid intelligence in healthy adults. Neuroimage 25:1175-1186.

Haier RJ, Colom R, Schroeder DH, Condon CA, Tang C, Eaves E, Head K (2009) Gray matter and intelligence factors: Is there a neuro-g? Intelligence 37:136-144.

Maguire EA, Gadian DG, Johnsrude IS, Good CD, Ashburner J, Frackowiak RS, Frith CD (2000) Navigation-related structural change in the hippocampi of taxi drivers. Proceedings of the National Academy of Sciences 97:4398-4403.

Takeuchi H, Taki Y, Sassa Y, Hashizume H, Sekiguchi A, Fukushima A, Kawashima R (2014) Regional gray matter volume is associated with empathizing and systemizing in young adults. PLoS ONE 9:e84782.

**Supplemental Figure Legends**

**Supplemental Fig. 1.** Comparison of the distribution of regional gray matter volume (rGMV) between science and humanities students and that of negative rGMV correlates of empathizing. The purple areas indicate significant clusters for a negative correlation between rGMV and empathizing. The purple regions are shown with *P* < 0.05, corrected for multiple comparisons at the non-isotropic adjusted cluster level with an underlying voxel level of *P* < 0.0025, uncorrected. The red areas indicate a non-significant tendency for a negative correlation between rGMV and empathizing. The results are shown with an underlying voxel level of *P* < 0.0025, uncorrected. The dark green areas indicate significant clusters for the difference in rGMV between science and humanities students. The dark green results are shown with *P* < 0.05, corrected for multiple comparisons at the non-isotropic adjusted cluster level with an underlying voxel level of *P* < 0.0025, uncorrected. The light green areas indicate a non-significant tendency for a difference in rGMV between science and humanities students. The results are shown with an underlying voxel level of *P* < 0.0025, uncorrected.

**Supplemental Fig. 2.** Comparison between the distribution of a weak tendency for a larger rGMV between science and humanities students and that for negative rGMV correlates of empathizing. The blue areas indicate a weak tendency for a negative correlation between rGMV and empathizing. The results are shown with an underlying voxel level of *P* < 0.05, uncorrected. The red areas indicate a non-significant tendency for the difference in rGMV between science and humanities students. The results are shown with an underlying voxel level of *P* < 0.05, uncorrected.

**Supplemental Fig. 1**

**
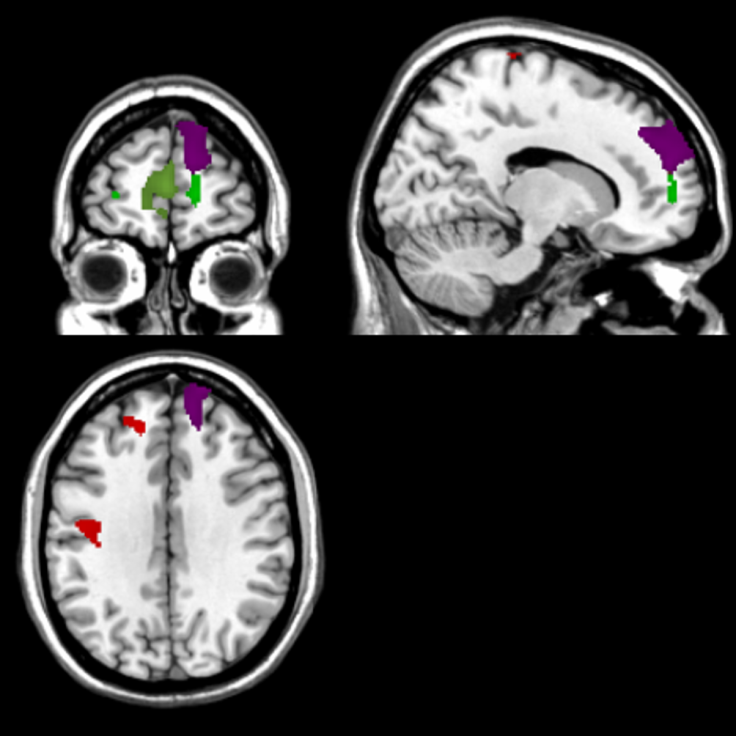
**

**Supplemental Fig. 2**

**
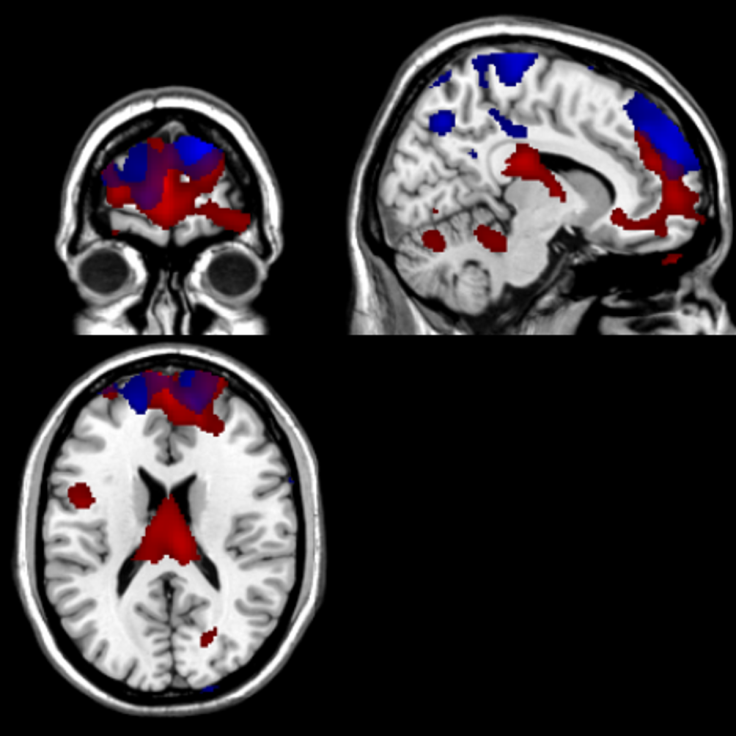
**
