# Supplementary figures and images for: Brain structures in the sciences and humanities
Source: Brain Struct Funct. 2014 Jul 31;220(6):3295–305. doi: 10.1007/s00429-014-0857-y (PMC4575694; doi:10.1007/s00429-014-0857-y)

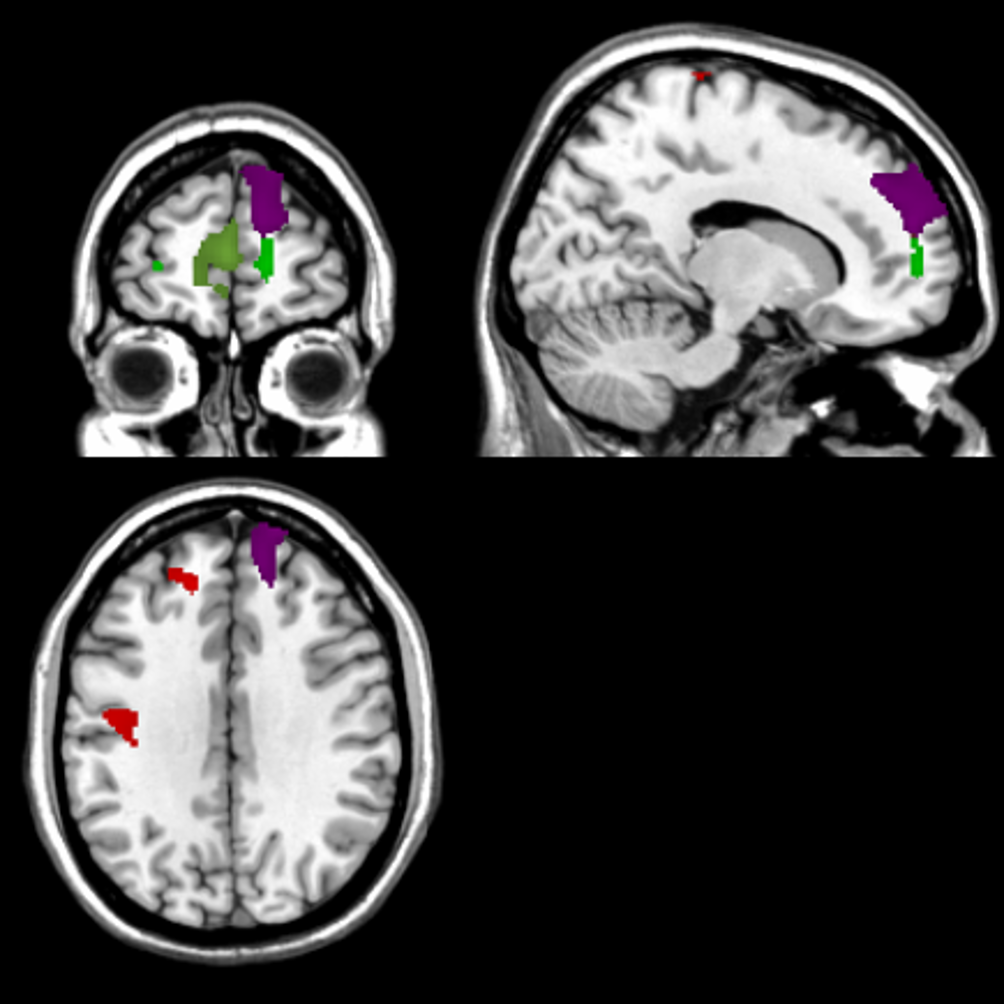

Supplement: Supplementary file 2 — Supplementary material 2 (TIFF 2953 kb) [file 429_2014_857_MOESM2_ESM.tif]

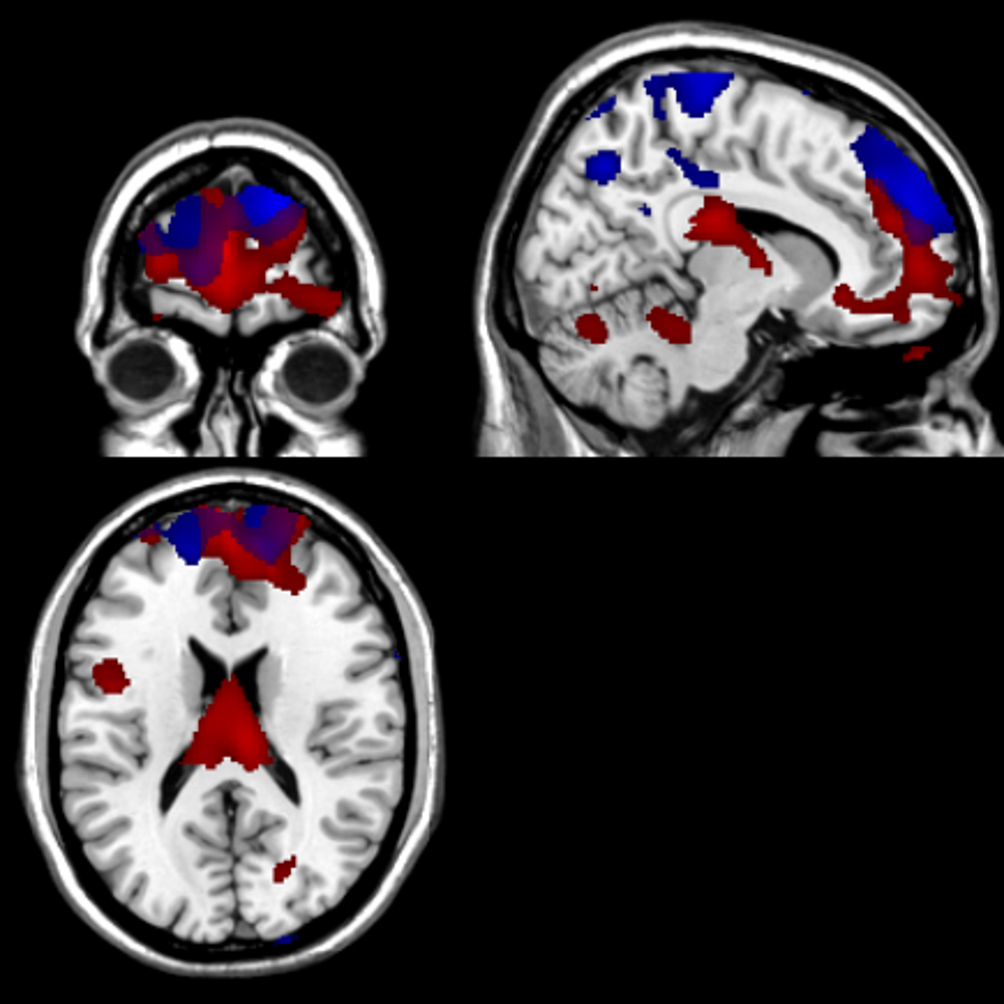

Supplement: Supplementary file 3 — Supplementary material 3 (TIFF 2953 kb) [file 429_2014_857_MOESM3_ESM.tif]
